# Correction: SERS-assisted characterization of cell biomass from biofilm-forming *Acinetobacter baumannii* strains using chemometric tools

**DOI:** 10.1039/d5ra90030a

**Published:** 2025-03-25

**Authors:** Arslan Yousaf, Muhammad Hafeez Ullah, Haq Nawaz, Muhammad Irfan Majeed, Nosheen Rashid, Abdulrahman Alshammari, Norah A. Albekairi, Arslan Ali, Munawar Hussain, Abu Bakar Salfi, Muhammad Aamir Aslam, Kinza Idrees, Allah Ditta

**Affiliations:** a Department of Chemistry, University of Agriculture Faisalabad Faisalabad 38000 Pakistan haqchemist@yahoo.com irfan.majeed@uaf.edu.pk; b Department of Chemistry, University of Education, Faisalabad Campus Faisalabad 38000 Pakistan; c Department of Pharmacology and Toxicology, College of Pharmacy, King Saud University Post Box 2455 Riyadh 11451 Saudi Arabia; d Institute of Microbiology, Faculty of Veterinary Sciences, University of Agriculture Faisalabad Faisalabad 38000 Pakistan aamir.aslam@uaf.edu.pk; e Institute for Experimental Molecular Imaging, RWTH Aachen University Hospital Aachen 52074 Germany

## Abstract

Correction for ‘SERS-assisted characterization of cell biomass from biofilm-forming *Acinetobacter baumannii* strains using chemometric tools’ by Arslan Yousaf *et al.*, *RSC Adv.*, 2025, **15**, 4581–4592, https://doi.org/10.1039/D4RA06267A.

The authors regret that an incorrect grant number was shown in the acknowledgements section of the published article. The corrected section should read:

The authors are thankful to the Researchers Supporting Project number (RSPD2025R1035), King Saud University, Riyadh, Saudi Arabia.

The Royal Society of Chemistry apologises for these errors and any consequent inconvenience to authors and readers.

